# An Unsupervised Machine Learning Clustering and Prediction of Differential Clinical Phenotypes of COVID-19 Patients Based on Blood Tests—A Hong Kong Population Study

**DOI:** 10.3389/fmed.2021.764934

**Published:** 2022-02-24

**Authors:** Kitty Yu-Yeung Lau, Kei-Shing Ng, Ka-Wai Kwok, Kevin Kin-Man Tsia, Chun-Fung Sin, Ching-Wan Lam, Varut Vardhanabhuti

**Affiliations:** ^1^Biomedical Engineering Programme, The University of Hong Kong, Hong Kong, Hong Kong SAR, China; ^2^Department of Diagnostic Radiology, Li Ka Shing Faculty of Medicine, The University of Hong Kong, Hong Kong, Hong Kong SAR, China; ^3^Department of Mechanical Engineering, The University of Hong Kong, Hong Kong, Hong Kong SAR, China; ^4^Department of Electrical and Electronic Engineering, The University of Hong Kong, Hong Kong, Hong Kong SAR, China; ^5^Department of Pathology, Li Ka Shing Faculty of Medicine, The University of Hong Kong, Hong Kong, Hong Kong SAR, China

**Keywords:** COVID-19, clinical phenotypes, laboratory test, machine learning, clustering

## Abstract

**Background:**

To better understand the different clinical phenotypes across the disease spectrum in patients with COVID-19 using an unsupervised machine learning clustering approach.

**Materials and Methods:**

A population-based retrospective study was conducted utilizing demographics, clinical characteristics, comorbidities, and clinical outcomes of 7,606 COVID-19–positive patients on admission to public hospitals in Hong Kong in the year 2020. An unsupervised machine learning clustering was used to explore this large cohort.

**Results:**

Four clusters of differing clinical phenotypes based on data at initial admission was derived in which 86.6% of the deceased cases were aggregated in one of the clusters without prior knowledge of their clinical outcomes. Other distinctive clinical characteristics of this cluster were old age and high concurrent comorbidities as well as laboratory characteristics of lower hemoglobin/hematocrit levels, higher neutrophil, C-reactive protein, lactate dehydrogenase, and creatinine levels. The clinical patterns captured by the cluster analysis was validated on other temporally distinct cohorts in 2021. The phenotypes aligned with existing literature.

**Conclusion:**

The study demonstrated the usefulness of unsupervised machine learning techniques with the potential to uncover latent clinical phenotypes. It could serve as a more robust classification for patient triaging and patient-tailored treatment strategies.

## Introduction

Coronavirus disease 2019 (COVID-19) is a respiratory disease caused by severe acute respiratory syndrome coronavirus (SARS-CoV-2). This novel coronavirus was first reported in Wuhan, China, in December 2019 and quickly spread worldwide (1). Belonging to the same coronavirus family as SARS-CoV and MERS-CoV, SARS-CoV-2 also exhibits a remarkable infectivity power (2). Immediate practices have been taken to allocate medical resources and plan for treatments. However, they might not be as effective as expected due to a lack of knowledge about SARS-CoV-2. A better understanding of the pathogenesis of COVID-19 and its different clinical phenotypes and risk groups is essential to address the immunopathology of the infection. The accumulated observational data on COVID-19 positive patients available to date serve as valuable resources. To probe this large amount of clinical data, supervised machine learning approaches have been applied to the diagnosis and prognosis of COVID-19, risk stratification, and the prediction of different outcomes (3–9). Unsupervised machine learning is an alternative approach that does not require specific labels. It avoids using preconceived knowledge or assumptions that may be subjected to biases and unknown confounders. To this end, unsupervised clustering techniques are often used for exploratory analysis to probe the underlying patterns within big data sets, enabling identification of latent clinical phenotypes and potentially deriving novel insights from the associated correlations. For example, it has been applied to derive the phenotypes of COVID-19 patients using electronic health record (EHR) data on admission of 6,000 COVID-19–positive adults at the Mount Sinai Health System in New York in the United States (10), 413 patients from an individual-level published study (11), and 213 patients in Wuhan Pulmonary Hospital (12). Hong Kong's health system is unique when dealing with the COVID-19 pandemic. Due to a government-wide policy, all COVID-19–positive patients were admitted to public hospitals regardless of their disease severity or symptoms. Therefore, the data in Hong Kong can capture the varying presentations of COVID-19. This study aims to use unsupervised clustering analysis to discover different phenotypic presentations across the disease spectrum of COVID-19 in Hong Kong based on demographics and laboratory information on admission to hospital.

## Materials and Methods

### Study Design and Participants

This study protocol was approved by institutional review boards in multiple hospitals across Hong Kong (see Supplementary Material for further details). Patient-informed consent was waived owing to the retrospective nature.

A retrospective search of patients' electronic records was conducted using the Hong Kong Hospital Authority Clinical Data Analysis and Reporting System (CDARS). It covered 42 hospitals across the territory in Hong Kong. Patients who were retrieved had a positive diagnosis of COVID-19 based on a reverse-transcriptase polymerase chain reaction (RT-PCR) test for SARS-CoV-2 fulfilling the testing criteria set by the Center for Health Protection, Department of Health, Government of Hong Kong SAR. The first cohort was retrieved from January 23 to December 31, 2020. The second cohort (used as a temporal validation set) was retrieved from January 1 to February 15, 2021.

Observational data, including demographics (age and sex), and 20 basic laboratory tests [white blood cell count (WBC), neutrophil count (NEUT), lymphocyte count (LYM), monocyte count (MON), hemoglobin (HGB), hematocrit (HCT), platelet (PLT), albumin (Alb), total bilirubin (TBIL), alanine aminotransferase (ALT), alkaline phosphatase (ALP), lactate dehydrogenase (LDH), creatine kinase (CK), urea, creatinine (Cr), C-reactive protein (CRP), sodium (Na), potassium (K), phosphate (P), and calcium (Ca)] were retrieved on the first day of admission (see Table 1). Blood tests with <50% of available data were excluded (13). Patients' comorbidities (19 systems) as specified by the international classification of disease (ICD-9) (14) were retrieved up to 3 days before that individual's admission to avoid including input of coding for the current admission. Mortality data was retrieved and set at 45 days after discharge for each patient to ensure that death, if it occurred, was likely related to COVID-19 and not from other causes. For more detailed information, please refer to Supplementary Table 1.

**Table 1:** Demographics and clinical characteristics of 7,606 COVID-19 positive patients.

**Patients characteristics** ** (*n* = 7,606)**	**Median (IQR)** or count (%)^*^	**Missing** ** count (%)**
**I. Demographics**		
Age (years)	47 (32–61)	0 (0%)
Sex (Males)	3,697 (48.6%)	0 (0%)
**II. Complete blood count**		
White blood cell count (^*^10^9^/L)	5.3 (4.3–6.6)	0 (0%)
Neutrophil count (^*^10^9^/L)	3.2 (2.4–4.3)	110 (1.4%)
Lymphocyte count (^*^10^9^/L)	1.3 (1.0–1.8)	110 (1.4%)
Monocyte count (^*^10^9^/L)	0.5 (0.4–0.7)	110 (1.4%)
Hemoglobin (g/dL)	13.7 (12.6–14.7)	0 (0%)
Hematocrit (L/L)	0.40 (0.37–0.43)	1 (<0.1%)
Platelet (^*^10^9^/L)	214 (173-264)	5 (<0.1%)
**III. Liver function**		
Albumin (g/L)	40.0 (37.2–43.5)	3 (<0.1%)
Total bilirubin (μmol/L)	7.9 (5.8–10.9)	8 (<0.1%)
Alanine aminotransferase (μ/L)	23.4 (16.0–36.0)	8 (<0.1%)
Alkaline phosphatase (μ/L)	66 (54–81)	8 (<0.1%)
**IV. Kidney function**		
Urea (mmol/L)	3.9 (3.1–4.8)	5 (<0.1%)
Creatinine (μmol/L)	69.1 (58.0–83.0)	5 (<0.1%)
**V. Inflammatory marker**		
C-reactive protein	0.4 (0.1–1.5)	872 (11.5%)
**VI. Electrolyte**		
Sodium (mmol/L)	138 (137–140)	5 (<0.1%)
Potassium (mmol/L)	3.8 (3.5–4.1)	31 (0.4%)
Phosphate (mmol/L)	1.04 (0.90–1.18)	2,778 (36.5%)
Calcium (mmol/L)	2.26 (2.18–2.34)	2,720 (35.8%)
**VII. Others**		
Lactate dehydrogenase (μ/L)	193.0 (165.0–235.0)	238 (3.1%)
Creatine kinase (μ/L)	91 (63–143)	818 (10.8%)

* *Decimal places were kept according to normal reference range*.

### Data Preparation and Preprocessing

Yeo–Johnson transformation was applied to provide multivariate normal distributions (15). Patients with more than 40% missing variables and inconsistent data were excluded (13). Multiple imputation *via* chained equations (MICE) (16) was adopted to handle missing data and produced the least biased estimation under the verified assumption missing at random (MAR) (17). Bayesian ridge regression was used to introduce variations. Ten iterations and ascending order of imputations were set and deemed to be sufficient (18).

Principal components analysis (PCA) was used to compress intrinsically correlated and dependent numerical variables and project them to low-dimension representations (see Supplementary Figures 1, 2). Ten principal components (PCs) were kept to preserve 80% of the variance (see Supplementary Figure 3).

### Model Training

*k*-prototype clustering (19) accounted for numerical data, and categorical data were used to probe the underlying clinical patterns of COVID-19–positive patients on admission. To select the number of clusters, the within-cluster sum of squared error (WCSS) was plotted from 1 to 10 clusters (see Supplementary Figure 4). According to the elbow method, four clusters were selected for the *k*-prototype model. The partitioning of three and five clusters was also examined (see Supplementary Figure 5).

### Model Evaluation

To evaluate the generated clusters, a surrogate prediction model was built to check if partitioning found by the *k*-prototype model was still preserved. A gradient-boosting decision tree model named LightGBM (20) was used to build the prediction model. It predicted COVID-19 prognosis based on electronic patient record data, and the performance was demonstrated (8). Eighty percent of features were used before training each decision tree to prevent overfitting. Missing data were ignored in the training. A separate cohort of 722 COVID-19 positive patients admitted from January 1 to February 15, 2021, were retrieved as a separate temporal validation set to assess the prediction (see Supplementary Table 2). Shapley additive explanation (SHAP) (21) was used to explain the feature importance of the prediction model. The data processing was conducted using Python version 3.8.5 (Python Software Foundation, Beaverton, USA) and available libraries.

### Statistical Analysis

Descriptive statistics were generated using SPSS version 26 (IBM Corp, Armonk, NY). Because the numerical data were not normally distributed, medians and interquartile ranges were reported. For categorical data, counts and percentages were reported. The level of missing data was also reported.

To compare the intercluster dissimilarity of numerical data, the central limit theorem (CLT) was applied, and parametric ANOVA was conducted. A *p* < 0.05 equates to statistical significance. Because the Levene's test of equality of error variances was statistically significant, equal variance across clusters was not assumed. Games–Howell *post-hoc* tests were performed for multiple comparisons. To compare the intercluster dissimilarity of categorical data, a chi-square test was conducted. Bonferroni *post-hoc* tests were performed for multiple comparisons. Interquartile range (IQR) and charts were reported and used for interpretation (22).

## Results

**Figure 1:**
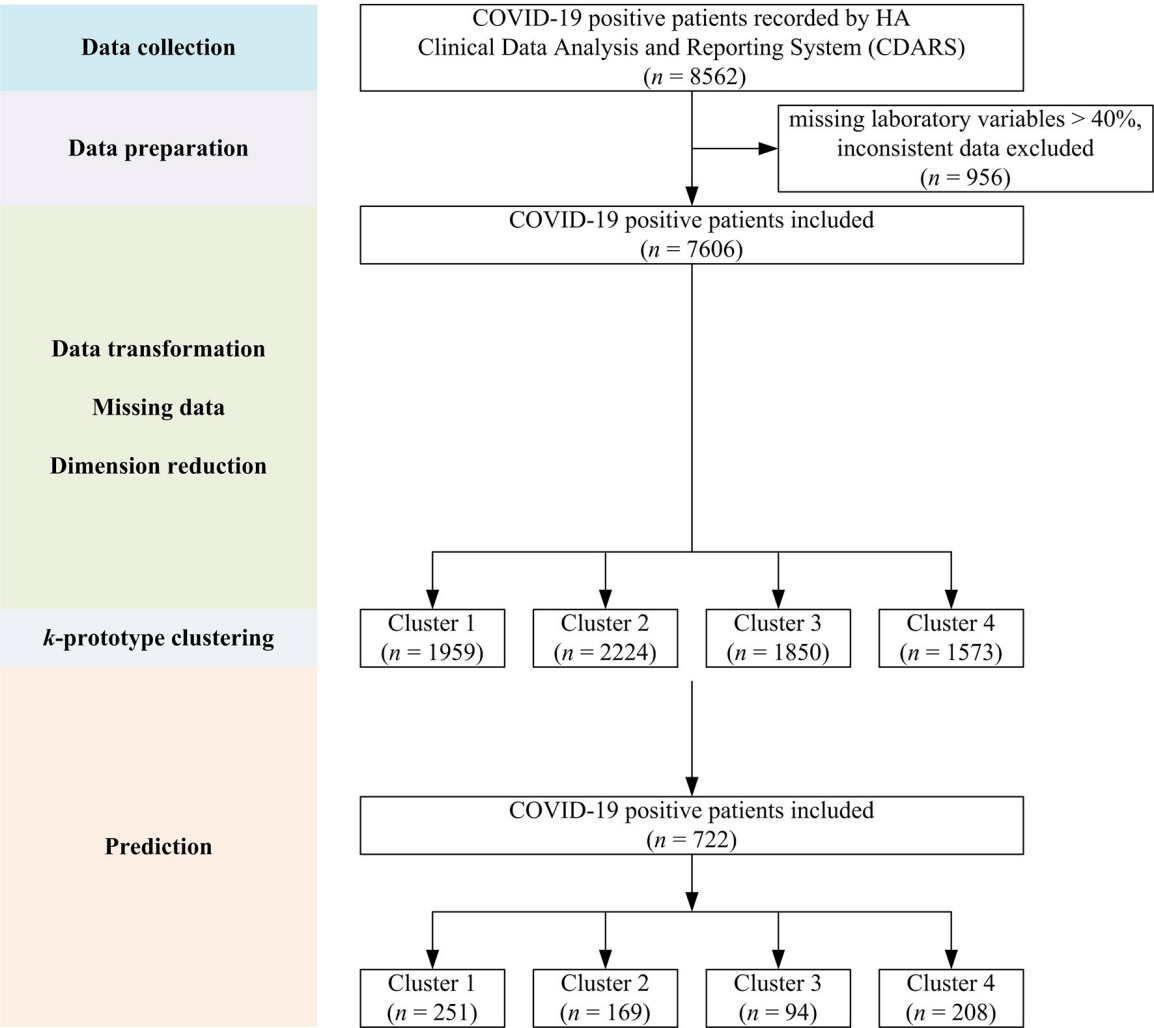
Flow chart showing the overall study design with data collection, preparation, model building, and prediction steps.

Initial data retrieval yielded a total of 8,562 patients. After data preparation to deal with missing variables and inconsistent data, a final number of 7,606 patients was kept (see Figure 1). The demographics and clinical characteristics are shown in Table 1.

**Figure 2:**
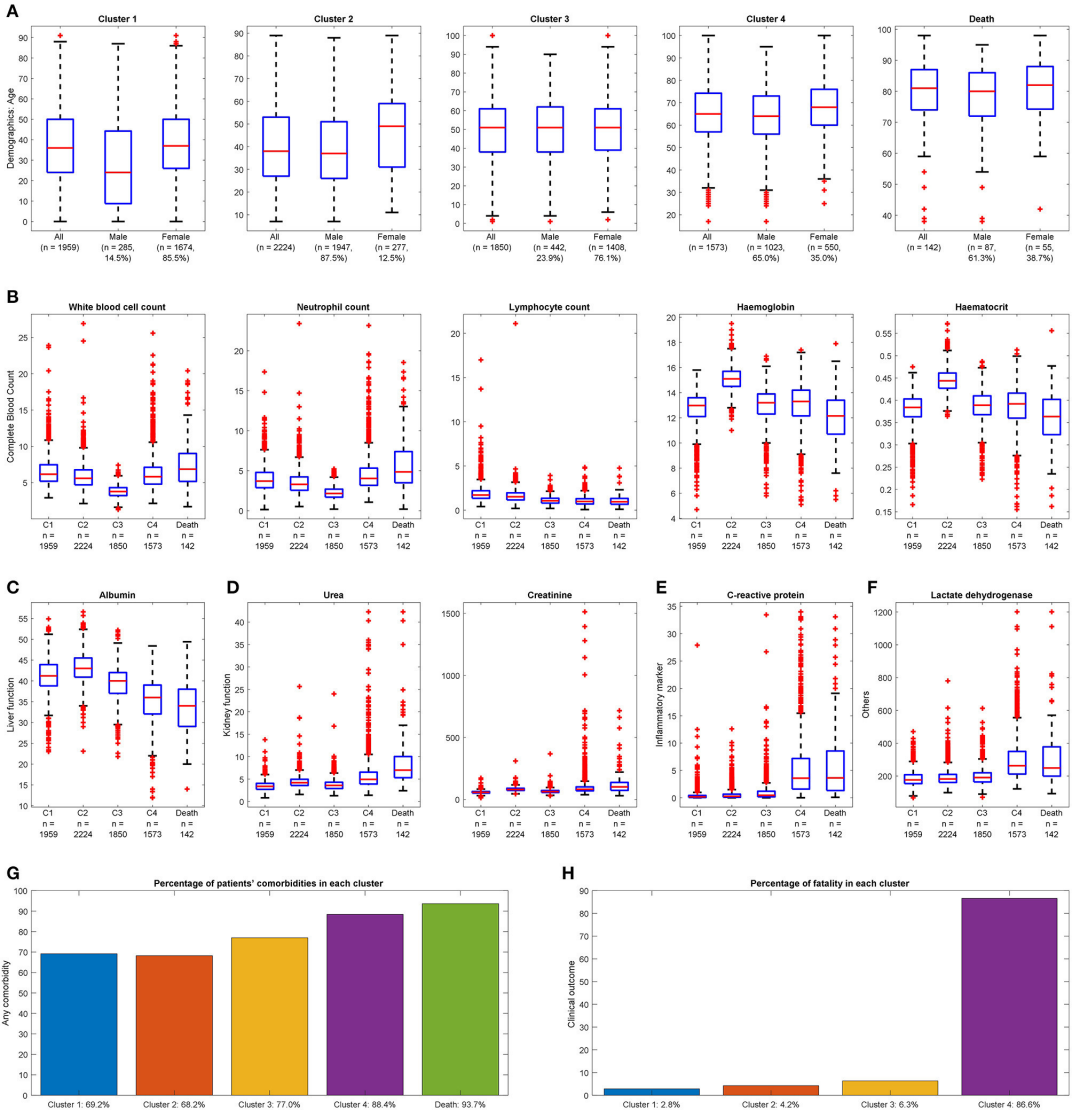
Distinguishable clinical characteristics of four clusters with deceased cases for reference. **(A)** Demographics: Cluster 4 has the highest mean age, and Cluster 2 has the highest proportion of males. Cluster 1 is the youngest group with the lowest proportion of males. **(B)** Complete blood count: Cluster 3 has the smallest range of white blood cell count; Cluster 4 has a larger range of neutrophil count and smallest range of lymphocyte count. Cluster 2 has the largest median for hemoglobin and hematocrit. **(C)** Liver function: Cluster 4 has the smallest mean value of albumin. **(D)** Kidney function: Cluster 4 has a higher range of urea and creatinine. **(E)** Inflammatory marker: Cluster 4 has a more extensive range of C-reactive protein. **(F)** Others: Cluster 4 has elevated lactate dehydrogenase. (A value of 1.5 times more than the IQR and away from the bottom or top of the box is considered an outlier.) **(G)** Any comorbidity: Cluster 4 has the most significant proportion with at least one comorbidity. **(H)** Clinical outcome: Highest fatality observed in Cluster 4.

Four clusters were identified using demographics and laboratory variables on admission. A separate analysis was done on the deceased cohort (see Table 2). The clusters were compared with one another. Each cluster was compared with the derived population and the most used normal reference range in the cohort. Each cluster was also compared with the deceased cases. A value was marked “high/low” if still within the reference range but considered close to the cutoff values. A value was marked “elevated/reduced” if it was outside the normal reference range (see Table 2, Figure 2; Supplementary Table 3).

**Table 2:** Demographics and clinical characteristics of four clusters and deceased cohort for comparison.

	**Characteristics** (unit; normal reference range^**^)	**Cluster 1** ** (*n* = 1,959)**	**Cluster 2** ** (*n* = 2,224)**	**Cluster 3** ** (*n* = 1,850)**	**Cluster 4** ** (*n* = 1,573)**	***p*-value**	**Deceased cohort**
		**Median** **(IQR) or count** **(within cluster %)**					
**I**.	**Demographics**						
	Age (years)	36a^***^ (24–50)	38b (27–53)	51c (38–61)	65d (57–75)	<0.001	81 (74–87)
	Sex (Males)	285a (14.5%)	1947b (87.5%)	442c (23.9%)	1023d (65.0%)		87 (61.3%)
**II**.	**Complete blood count**						
	White blood Cell count (*10^9^/L; 3.7–9.2)	6.2a (5.2–7.5)	5.6b (4.7–6.8)	3.8c (3.2–4.3)	5.8d (4.8–7.1)	<0.001	6.9 (5.1–9.0)
	Neutrophil count (*10^9^/L; 1.7–5.8)	3.7a (2.9–4.8)	3.3b (2.6–4.2)	2.2c (1.7–2.7)	4.0d (3.2–5.3)	<0.001	4.9 (3.5–7.4)
	Lymphocyte count (*10^9^/L; 1.0–3.1)	1.7a (1.3–2.2)	1.5b (1.2–2.0)	1.1c (0.8–1.3)	1.0d (0.7–1.3)	<0.001	1.0 (0.7–1.3)
	Monocyte count (*10^9^/L; 0.1–0.8)	0.5a (0.4–0.7)	0.6b (0.4–0.7)	0.4c (0.3–0.5)	0.5a (0.4–0.7)	<0.001	0.6 (0.3–0.8)
	Hemoglobin (g/dL; 11.7–14.9)	13.0a (12.1–13.6)	15.1b (14.5–15.7)	13.2c (12.3–13.9)	13.3c (12.1–14.2)	<0.001	12.2 (10.7–13.4)
	Hematocrit (L/L; 0.35–0.45)	0.38a (0.36–0.40)	0.44b (0.43–0.46)	0.39c (0.37–0.41)	0.39c (0.36–0.42)	<0.001	0.36 (0.32–0.40)
**III**.	**Liver function**						
	Albumin (g/L; 35.0–52.0)	41.2a (38.8–43.9)	43.0b (40.9–45.5)	40.0c (37.0–42.0)	36.0d (32.0–39.0)	<0.001	34.0 (29.0–38.1)
	Total bilirubin (μmol/L; 5.0–21.0)	7.0a (5.0–9.7)	9.5b (7.0–12.9)	6.6c (5.0–9.0)	8.2d (6.2–11.3)	<0.001	8.2 (6.0–11.6)
	Alanine aminotransferase (μ/L; 0.0–34.4)	18.0a (13.1–27.0)	29.0b (20.0–45.0)	20.0a (14.0–28.5)	29.0b (20.0–44.0)	<0.001	20.8 (14.0–33.2)
	Alkaline phosphatase (μ/L; 30–120)	67a (54–87)	69b (58–82)	60c (50–73)	67b (56–84)	<0.001	72 (59–103)
**IV**.	**Kidney function**						
	Urea (mmol/L; 2.8–8.1)	3.4a (2.7–4.1)	4.2b (3.6–5.0)	3.6c (2.9–4.3)	4.9d (3.9–6.6)	<0.001	7.0 (5.3–10.0)
	Creatinine (μmol/L; 49.0–90.0)	57.0a (50.8–64.0)	80.0b (72.0–90.0)	72.0c (62.8–84.0)	82.0d (69.0–101.0)	<0.001	101.1 (73.0–137.7)
**V**.	**Inflammatory marker**						
	C–reactive protein (mg/dL; 0.0–5.0)	0.2a (0.1–0.4)	0.3b (0.1–0.7)	0.5c (0.2–1.2)	3.6d (1.6–7.2)	<0.001	3.6 (1.3–8.6)
**VI**.	**Electrolyte**						
	Sodium (mmol/L; 136–145)	139a (137–140)	139a (138–140)	139b (137–140)	136c (133–138)	<0.001	136 (133–140)
	Potassium (mmol/L; 3.4–4.8)	3.8a (3.5–4.0)	3.9b (3.6–4.1)	3.7c (3.4–3.9)	3.8a (3.5–4.1)	<0.001	4.0 (3.7–4.3)
	Phosphate (mmol/L; 0.88–1.45)	1.11a (0.98–1.27)	1.07b (0.95–1.20)	1.02c (0.89–1.15)	0.95d (0.83–1.09)	<0.001	1.08 (0.90–1.23)
	Calcium (mmol/L; 2.15–2.55)	2.31a (2.25–2.38)	2.33a (2.26–2.39)	2.22b (2.16–2.28)	2.17c (2.10–2.24)	<0.001	2.14 (2.06–2.25)
**VII**.	**Others**						
	Lactate dehydrogenase (μ/L; 0.0–246.4)	176.5a (154.0–208.0)	182.2b (161.0–210.0)	191.0c (164.0–220.0)	263.0d (212.0–350.0)	<0.001	249.0 (199.1–378.8)
	Creatine kinase (μ/L; 39–308)	67a (49–91)	104b (76–151)	82c (59–120)	141d (85–258)	<0.001	131 (71–250)

** *Most used normal reference range*.

*** *Each subscript letter denotes a cluster whose column proportions do not differ significantly from each other at the 0.05 significance level. The clusters with different letter are significantly different from each other at the 0.05 significance level*.

### General Characteristics

The size of the four clusters were cluster 1: *n* = 1,959, cluster 2: *n* = 2,224, cluster 3: *n* = 1,850, and cluster 4: *n* = 1,573. Clusters 1 and 2 had the youngest median age [cluster 1: 36 (IQR: 24–50), cluster 2: 38 (IQR: 27–53)]. Increasing median age was observed from cluster 1 to 4. Clusters 2 and 4 had a greater proportion of males [cluster 2: *n* = 1,947 (87.5%), cluster 4: *n* = 1,023 (65.0%)]. The lymphocyte count was in the low value of the reference range in four clusters [cluster 1: 1.7 (IQR: 1.3–2.2), cluster 2: 1.5 (IQR: 1.2–2.0), cluster 3: 1.1 (IQR: 0.8–1.3), cluster 4: 1.0 (IQR: 0.7–1.3), normal reference range: 1.0–3.1]. Most of the indicators for liver function, including total bilirubin and alanine phosphatase levels, and for electrolytes, including sodium and potassium, were in the normal reference range for four clusters. Specific cluster analysis was discussed as follows.

### Cluster 1

Of COVID-19–positive patients, 25.4% (*n* = 1,959) were aggregated in cluster 1. Cluster 1 was characterized by the youngest median age and the highest proportion of females (85.5%) within the cluster. The laboratory test results were unremarkable.

### Cluster 2

Of COVID-19–positive patients, 28.9% (*n* = 2,224) were aggregated in cluster 2. Cluster 2 was characterized as the largest cluster with the highest proportion of males (87.5%) within the cluster. Hemoglobin, hematocrit, and creatinine levels were observed in the higher range of the reference values.

### Cluster 3

Of COVID-19–positive patients, 24.0% (*n* = 1,850) were aggregated in cluster 3. Cluster 3 was characterized by a higher proportion of females (76.1%) within the cluster. White blood cell counts were observed in the lower range of the reference values.

### Cluster 4

Of COVID-19–positive patients, 20.4% (*n* = 1,573) were grouped in cluster 4. Significantly, cluster 4 captured 123 out of 142 (86.6%) deceased cases. Cluster 4 was characterized by being the smallest cluster with the oldest median age and a higher proportion of males. In terms of blood tests, hemoglobin, hematocrit, lymphocyte, and albumin levels were observed in the lower range. Neutrophil, urea, and C-reactive protein were observed in the higher range of the reference values. Lactate dehydrogenase was elevated. Cluster 4 also had the highest comorbidity scores with triple the rate of immunity disorders and diseases of the circulatory systems; double the rate of diseases of the nervous systems; and a higher proportion of disease of the digestive system, genitourinary system, skin and subcutaneous tissue musculoskeletal system, other symptoms, injuries, and morphology of neoplasms compared with the other clusters. Cluster 4 captured almost all clinical characteristics of the deceased cases with the deceased cases having an even older median age and higher comorbidity scores.

### Deceased Cohort

There were 142 deceased cases in 7,606 censored COVID-19–positive patients. The deceased cohort was characterized by an old median age and a higher proportion of males. In terms of blood tests, lymphocyte, platelet, and albumin were observed in the lower range of the reference values. Urea and creatinine were observed in the higher range of the reference value. C-reactive protein and lactate dehydrogenase, although still in the reference range, were comparable to that of cluster 4 appearing higher than the other clusters. The comorbidity scores of the deceased cohort were high. There was a high degree of concordance with cluster 4.

### Evaluation

Cluster analysis was also applied to a separate temporal validation set with the results of seven out of eight (87.5%) deceased cases being captured by cluster 4.

**Figure 3:**
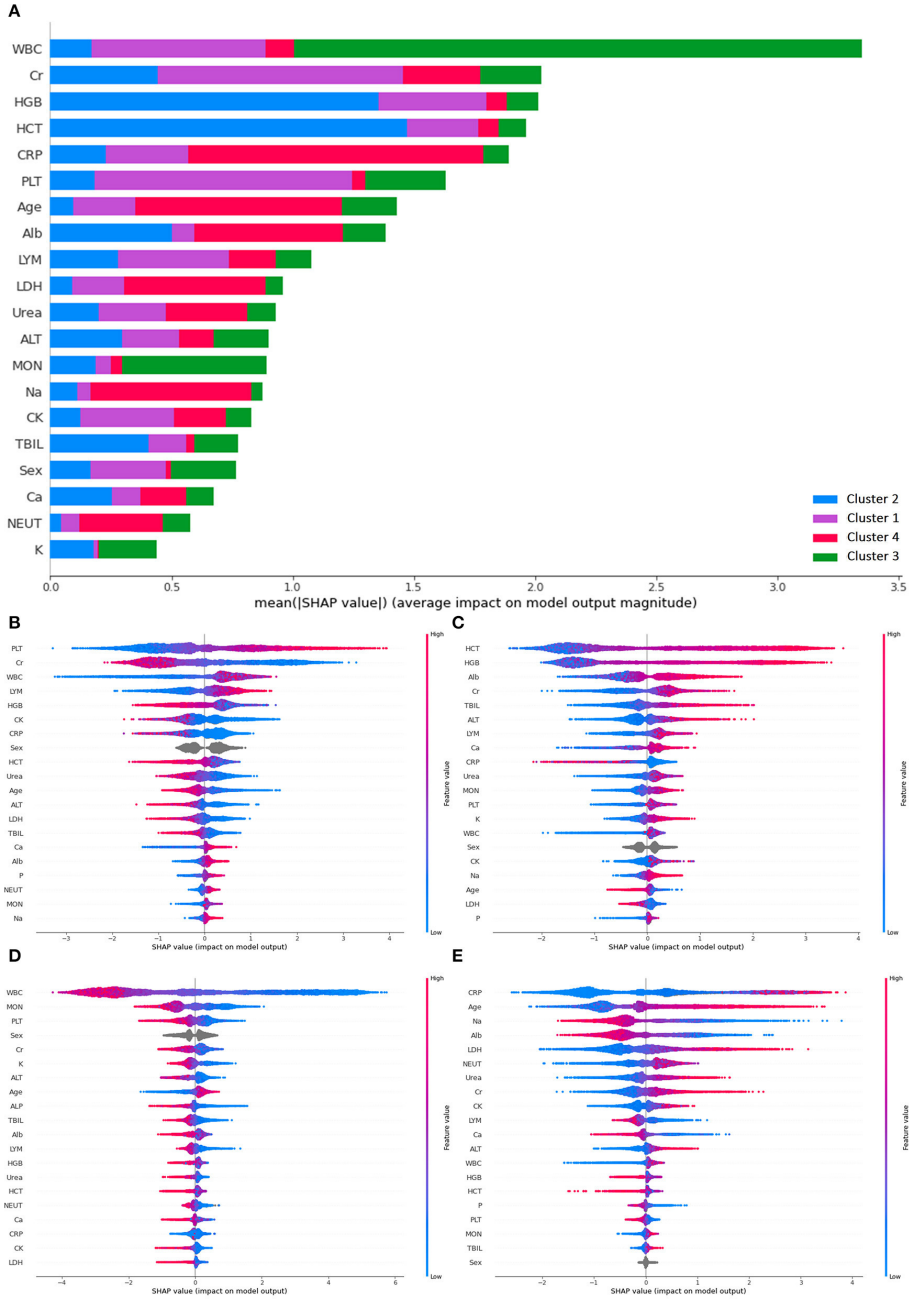
SHAP plots demonstrating differential importance of different features and clusters. **(A)** Mean SHAP value of the prediction, **(B)** SHAP value for cluster 1, **(C)** SHAP value for cluster 2, **(D)** SHAP value for cluster 3, **(E)** SHAP value for cluster 4. ALT, Alanine aminotransferase; Alb, albumin; ALP, alkaline phosphatase; Ca, calcium; CRP, C-reactive protein; CK, creatine kinase; Cr, creatinine; HCT, hematocrit; HGB, hemoglobin; LDH, lactate dehydrogenase; LYM, lymphocyte count; MON, monocyte count; NEUT, neutrophil count; P, phosphate; PLT, platelet; K, potassium; Na, sodium; TBIL, total bilirubin; WBC, white blood cell.

To further verify if criteria generated by *k*-prototype clustering were properly followed, SHAP was used to determine the feature importance used in the classification (see Figure 3). Overall, white blood count has the highest mean SHAP value for the prediction, followed by creatinine, hemoglobin, hematocrit, C-reactive protein, and platelet. High platelet and low creatinine were most important in classifying patients into cluster 1. Elevated hemoglobin and hematocrit were most important in classifying patients into cluster 2. Reduced white blood cell count was the most important in classifying patients into cluster 3. High C-reactive protein and old age were most important in classifying patients into cluster 4.

## Discussion

Unsupervised clustering was used to probe the latent phenotypes of 7,606 COVID-19–positive patients in Hong Kong across 2020. Based on age, sex, and 20 laboratory blood tests on admission, one of the four generated clusters aggregated 86.6% of deceased cases without prior information of their clinical outcomes. The clinical characteristics of this cluster, including the oldest median age; highest aggregated comorbidity; and the laboratory tests of hemoglobin, hematrocrit, and lymphocyte were observed to be in the lower range of normal, whereas neutrophil, C-reactive protein, and lactate dehydrogenase were observed to be in the higher range of normal. Findings are comparable with poor prognostic features based on contemporaneous literature. Notably, when applied to a separate validation cohort, cluster 4 was still able to identify seven out of eight (87.5%) deceased cases. A potential clinical utility may be to call for early medical attention and resource to patients that belong to cluster 4 at the initial diagnosis at hospitalization. The clinical characteristics of cluster 4 and deceased cases in this study aligned with previous findings (23–25). First, old age reflects a weaker immune system to fight against pathogens, including pneumonia (26). It is a well-established risk factor correlated with chronicity and comorbidity in COVID-19 prognosis (10, 27). On the other hand, increased neutrophil count and high neutrophil-to-lymphocyte ratio can be elicited by deep airway and alveolar damage. They represent an acute inflammatory response and are indicators of a poor prognosis and higher disease severity for COVID-19 (28–31). In line with cluster 4 and the deceased cases in this study, at an early stage of COVID-19, lymphocyte counts typically decrease, whereas white blood cell count may or may not decrease (32). In addition, patients with severe COVID-19 outcomes have exhibited abnormally high C-reactive protein levels, lactate dehydrogenase, and neutrophils, implying an inflammatory response and severe pneumonia (33). Although C-reactive protein level is not affected by physical status, age, and sex (34), it may be used to early diagnose severe pulmonary disease secondary to bacterial infection (35).

**Figure 4:**
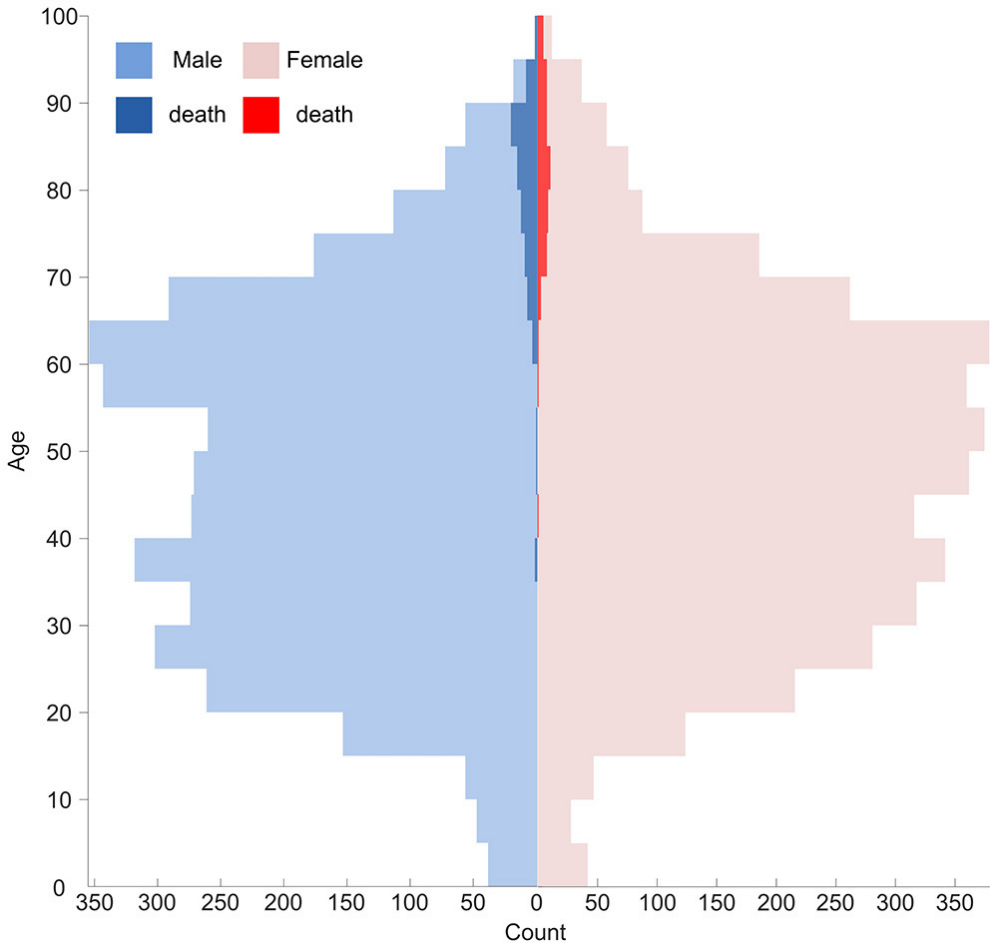
Population pyramid of the different age and sex with mortality subgroup.

A greater proportion of males than females were infected with COVID-19 in contemporary literatures as well as MERS-CoV and SARS-CoV infection (24, 36, 37). It can be a cofounder in evaluating the sex-based difference in the susceptibility of COVID-19 (38). In this retrospective study, the proportion of males and females was roughly equal across all age groups (see Figure 4) on admission. No significant difference in disease prevalence existed between males and females. Under this condition, a higher proportion of males was still observed in cluster 4 (65.0%) and deceased cases (61.3%). It indicates being male as a risk factor for mortality (37, 39, 40). This reduced disease susceptibility of females is hypothesized to be related to the major roles of the X chromosome and sex hormones in innate and adaptive immunity (41). Nonetheless, this correlation needs further exploration and investigation.

The complications of COVID-19 include severe pneumonia, septic shock, acute respiratory distress syndrome (ARDS), and multiple organ failure (31, 42). Although lung has been the primary organ involved in the infection, elevated creatinine and urea levels were also detected in cluster 4 and in the deceased cases, which implies that impaired renal function was a potential early indicator of poor prognosis. Additionally, a low albumin level, reflective of liver synthetic function, was also observed. These abnormal laboratory variables imply that indicators for early multiorgan failure were important and is likely a reflection of systemic inflammatory response. For the reduced hemoglobin and hematocrit levels detected in cluster 4 and the deceased, a prior study suggests that SARS-CoV-2 may attack the heme on 1-beta chain of hemoglobin through CD147, and CD26, ACE2 receptors or by simulating hepcidin to increase tissue ferritin, block ferroprotein, causing the iron deficiency, and thus lower hemoglobin level (43).

In previous clustering analysis (10–12), both three and four clusters have been identified, which were similar to the number chosen by this study. Previous clusters have also found some correlations for a poor prognosis, such as old age and high comorbidity scores (12, 28), being male, high lymphocytes high neutrophil count (11), and albumin level (12). In respect to the clinical outcomes, our study shows the greatest power to differentiate a cluster by aggregating 86.6% deceased cases and capturing most of their clinical characteristics and correlations.

There were several key strengths to our study. First, a large sample size of 7,606 COVID-19–positive patients was included. This adds to greater statistical power and generalizability. The data were collected from a single integrated EHR system fully covering the Hong Kong population, meaning the clinical data set is representative of this cohort. Second, all patients that were tested positive despite mild or no symptoms were nevertheless hospitalized in Hong Kong. Our study is unique capturing a wide spectrum of clinical severity. On the contrary, most other countries only tend to admit patients with severe symptoms to the hospitals. Third, we validated our clusters on a separate temporal validation with further feature explanation using SHAP. It was demonstrated that criteria from PCA-based *k*-prototype clustering were correctly captured, used, and inferred. Granular analysis of these distinct clusters should give a better understanding of different clinical representations of COVID-19 on admission. Besides this, delineating interactions between specific chronicity and poor prognosis regarding different clinical groups can help understand the mechanism of COVID-19.

There were a few limitations to our study. First, for a retrospective study using observational data, the sampling was not random. There may be the potential of selection and recall bias. Although all age groups were represented, they were not evenly distributed. The results and interpretations will be best applied to the Hong Kong cohort with race dominated by patients of Chinese ethnicity although there were several imported cases of different ethnic origins. Owing to a lack of accurate data on ethnicity, we could not perform further sub-analysis. Second, as with any large-scale studies covering multiple hospitals, there may be different standards and measurement protocols. It was observed that the normal reference ranges of laboratory variables varied mildly across institutions and according to age and sex. Because no significant discrepancy was detected, the data were put together directly using their original values. Although a linear mapping could have been used to reduce the systematic noise, variations due to age and sex need to be further distinguished. Finally, some potential important clinical and laboratory data with more than 50% of missing data were excluded from the study. They include BMI, blood pressure, oxygen saturation, D-Dimer, need for assisted ventilation, ITU admission, etc., which are potentially useful for severity assessment and prognostication. These may additionally aid prediction and more accurate allocation of medical resources in the future. Owing to a lack of testing or accurate documentations, these were not able to be included in this large scale multi-institutional study. These variables are recommended to be documented or performed in the future. However, they were not routinely recorded nor taken in usual clinical practice in Hong Kong at the time of the study period.

## Conclusion

Unsupervised clustering was used to probe the latent phenotypes of 7,606 COVID-19–positive patients in Hong Kong across the year 2020. Based on age, sex, and 20 laboratory variables on admission, one of the four generated clusters aggregated 86.6% deceased cases without prior knowledge of their clinical outcomes. Further understanding of the different COVID-19 clinical phenotypes may pave the way for more individualized patient risk stratification and treatment.

## Data Availability Statement

Data can be provided upon reasonable request and inquiries can be directed to the corresponding author

## Ethics Statement

The studies involving human participants were reviewed and approved by Cluster Research Ethics Committee/Institutional Review Board (REC/IRB). Written informed consent for participation was not required for this study in accordance with the national legislation and the institutional requirements.

## Author Contributions

KL, K-SN, and VV were involved in study conception, statistical analysis, and drafting of the initial version of the manuscript. K-SN and VV were involved in data collection. K-WK, KT, C-FS, C-WL, and VV supervised the work and offered significant intellectual contribution. All authors discussed the data analysis, results, and approved the final version.

## Conflict of Interest

The authors declare that the research was conducted in the absence of any commercial or financial relationships that could be construed as a potential conflict of interest.

## Publisher's Note

All claims expressed in this article are solely those of the authors and do not necessarily represent those of their affiliated organizations, or those of the publisher, the editors and the reviewers. Any product that may be evaluated in this article, or claim that may be made by its manufacturer, is not guaranteed or endorsed by the publisher.
