# Supplementary material for: An Unsupervised Machine Learning Clustering and Prediction of Differential Clinical Phenotypes of COVID-19 Patients Based on Blood Tests—A Hong Kong Population Study
Source: Front Med (Lausanne). 2022 Feb 24;8:764934. doi: 10.3389/fmed.2021.764934 (PMC8907521; doi:10.3389/fmed.2021.764934)
Supplement: Supplementary file 1 [file Data_Sheet_1.docx]

Supplementary Material

**Materials and Methods**

This study protocol was approved by multi-institutional review boards in multiple hospitals across Hong Kong: HKU/Hong Kong West Cluster Research Ethics Committee (Ref. UW 20-291), Hong Kong East Cluster Research Ethics Committee (HKECREC-2020-012), Kowloon Central/Kowloon East Cluster Research Ethics Committee (KC/KE-20-0052/ER-3), Kowloon West Cluster Research Ethics Committee (Ref. KW/EX-20-065), CUHK/New Territories East Cluster Clinical Research Ethics Committee (Ref. 2020.216), and New Territories West Cluster Research Ethics Committee (NTWC/REC/20048).

***k*-prototype Clustering**

In *k*-prototype Clustering, the cost function $E_{l}$ for cluster $l$ was calculated as the following. It uses Euclidean distance to calculate the cost function for numerical data $E_{l}^{u}$ and modified simple matching dissimilarity $\delta(x_{ij}^{c},\mathrm{o}_{lj}^{c})$ weighted by the standard deviation of numerical variables $\gamma_{l}$ to calculate the cost function for categorical data $E_{l}^{c}$, where $n$ is the number of the $\mathrm{x}$ is the data point, $o$ is the centroid of the cluster. When the category matches, a distance of $1-\omega(x_{ij}^{c},l)$ is used, where $f(x_{ij}^{c}|c_{l})$ is the frequency of $x_{ij}^{c}$ in cluster $l$, $|c_{l}|$ is the size of cluster $l$, and $f(x_{ij}^{c}|D)$ is the frequency of the entire dataset.

$$\begin{matrix} E_{l}=E_{l}^{u}+E_{l}^{c} \\ =\sum_{i=1}^{n} y_{il}\sum_{j=1}^{m_{u}} (\mathrm{x}_{ij}^{u}-o_{lj}^{u})^{2}+\gamma_{l}\sum_{i=1}^{n} y_{il}\sum_{j=1}^{m_{c}} \delta(\mathrm{x}_{ij}^{c}-o_{lj}^{c}). \end{matrix}$$

$\delta(x_{ij}^{c},\mathrm{o}_{lj}^{c})=\left\{\begin{matrix} 1-\omega(x_{ij}^{c},l), & x_{ij}^{c}=z_{lj}^{c} \\ 1, & x_{ij}^{c}\neq z_{lj}^{c} \end{matrix}\right.,$ where

$$\omega(x_{ij}^{c},l)=\frac{f(x_{ij}^{c}|c_{l})}{|c_{l}|\cdot f(x_{ij}^{c}|D)}.$$

Regarding the scatterplot along age and hemoglobin (HGB) (see Supplementary Figure 5), when the number of clusters increases from three to four, a new cluster is divided mainly from cluster 1 and partly from cluster 3. When the number of clusters increases from four to five, a subcluster is divided from cluster 4. The partitioning is in line, and no arbitrary pattern arises. Four clusters are approved to be the starting point when deceased cases are bonded by one of the clusters without being divided further. Therefore, further analysis was done on four clusters.

# Supplementary Figures and Tables.

Supplementary Figure 1. Correlation matrix of numerical variables after transformation and imputations; Supplementary Figure 2. Loading matrix; Supplementary Figure 3. Cumulated percentage of explained variance vs. PCs; Supplementary Figure 4. Numbers of cluster vs. WCSS for *k*-prototype model; Supplementary Figure 5. The paritioning and distribution along age and haemoglobin for (A) three clusters (B) four clusters, and (C) five clusters.


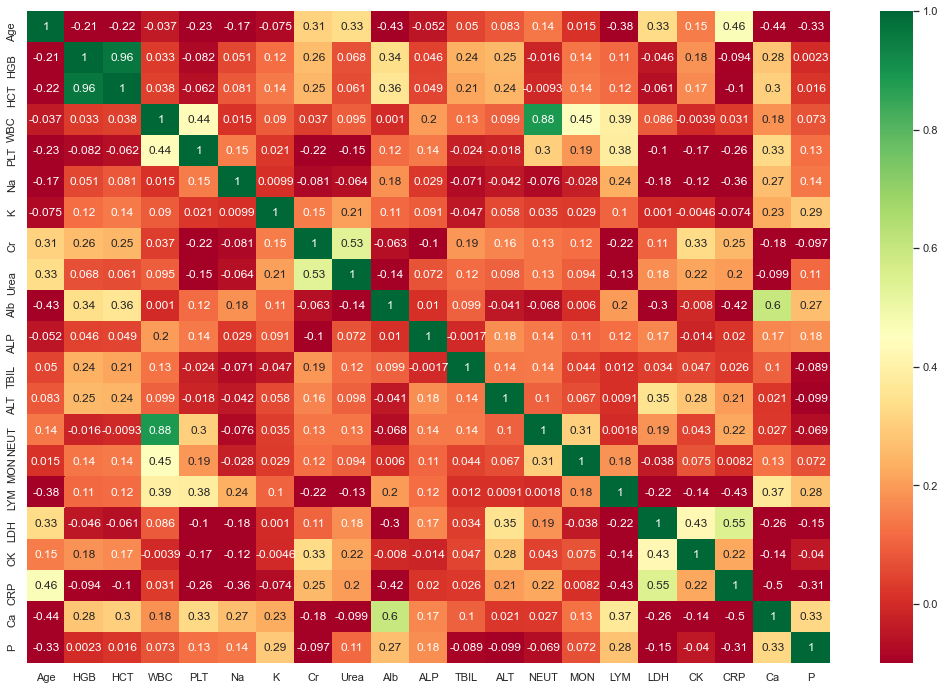


Supplementary Figure 1. Correlation matrix of numerical variables after transformation and imputations


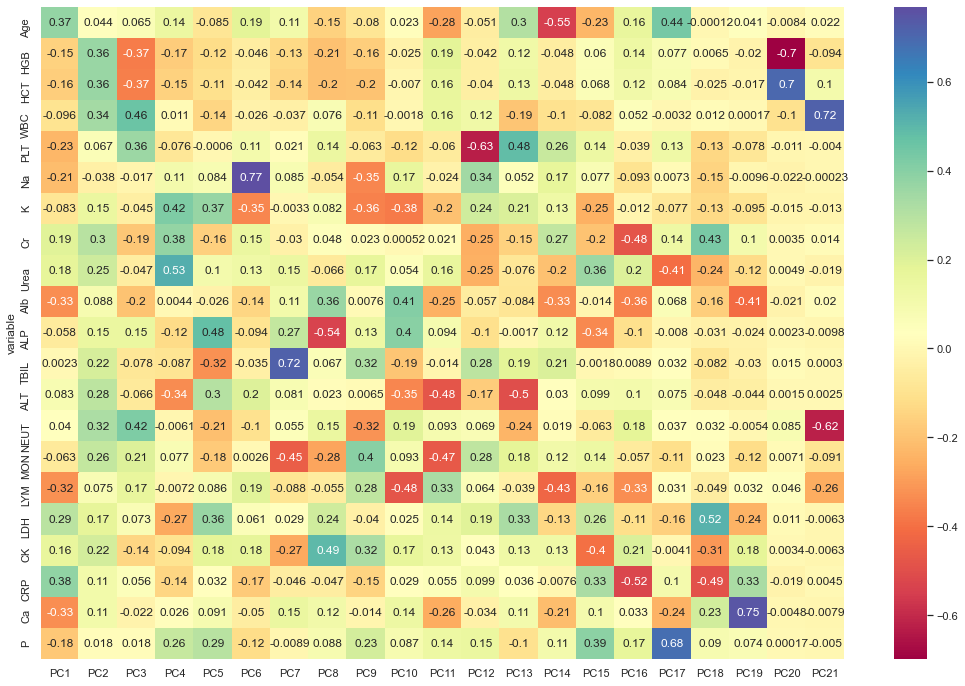
 Supplementary Figure 2. Loading matrix


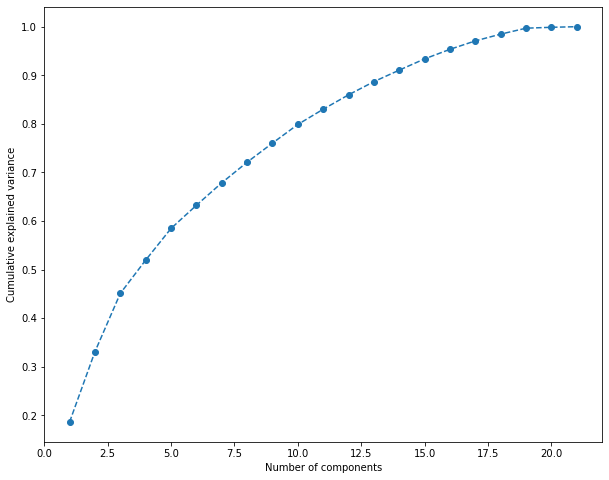


Supplementary Figure 3. Cumulated percentage of explained variance vs. PCs


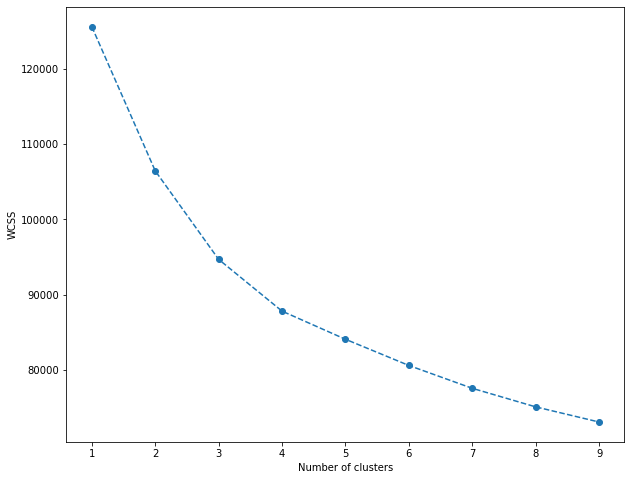


Supplementary Figure 4. Numbers of cluster vs. WCSS for *k*-prototype model

**
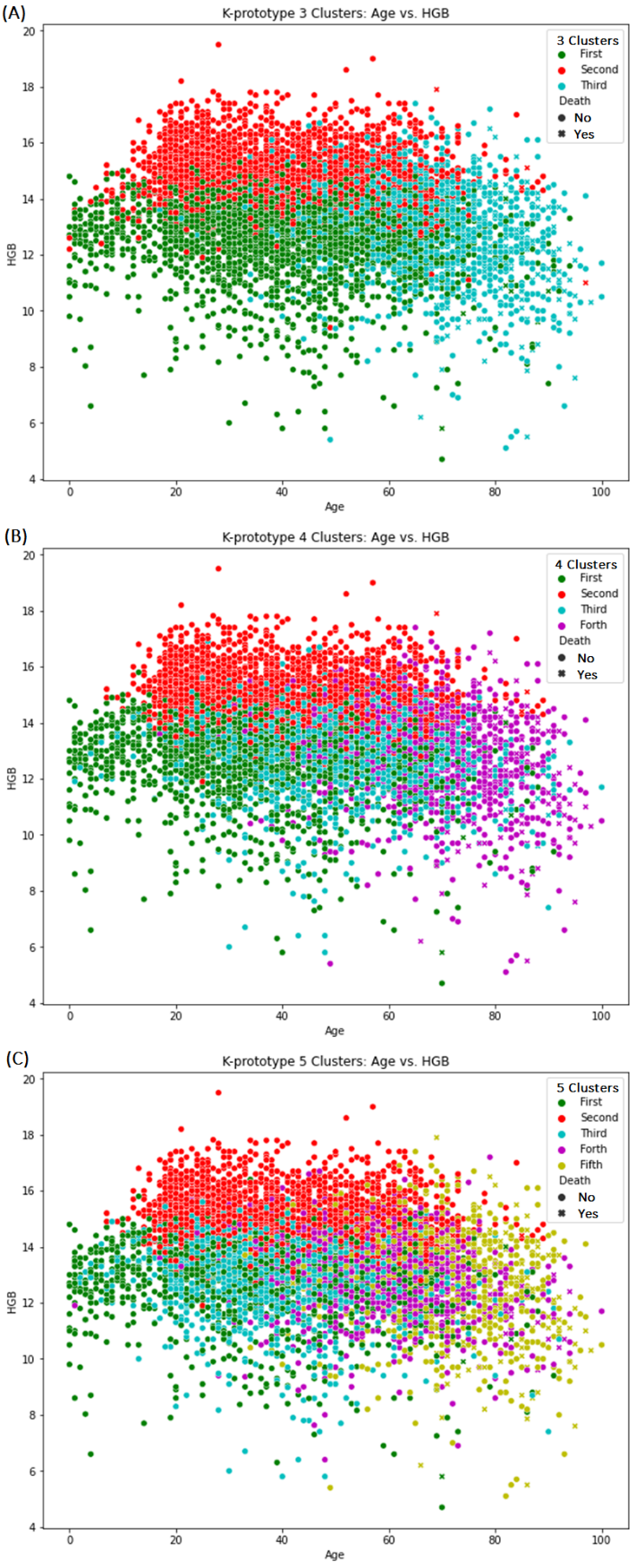
**

Supplementary Figure 5. The paritioning and distribution along age and haemoglobin for (A) three clusters (B) four clusters, and (C) five clusters

Supplementary Table 1. Comorbidities and clinical outcomes of 7606 COVID-19 positive patients

| **Patients (*n* = 7606) Characteristics** | | **Median (IQR) or count (%)** | |
| --- | --- | --- | --- |
| 1. **Comorbidities*** | |  | |
| Any | | 5687 (74.8%) | |
| Infectious and Parasitic Diseases | | 1781 (23.4%) | |
| Neoplasms | | 543 (7.1%) | |
| Endocrine, Nutritional and Metabolic Diseases, and Immunity Disorders | | 1396 (18.4%) | |
| Diseases of the Blood and Blood-forming Organs | | 343 (4.5%) | |
| Mental Disorders | | 682 (9.0%) | |
| Diseases of the Nervous System and Sense Organs | | 1043 (13.7%) | |
| Diseases of the Circulatory System | | 1565 (20.6%) | |
| Diseases of the Respiratory System | | 2613 (34.4%) | |
| Diseases of the Digestive System | | 1600 (21.0%) | |
| Diseases of the Genitourinary System | | 1236 (16.3%) | |
| Complications of Pregnancy, Childbirth, and the Puerperium | | 681 (9.0%) | |
| Diseases of the Skin and Subcutaneous Tissue | | 653 (8.6%) | |
| Diseases of the Musculoskeletal System and Connective Tissue | | 1200 (15.8%) | |
| Symptoms, Signs, and Ill-defined Conditions | | 2779 (36.5%) | |
| Injury and Poisoning | | 2195 (28.9%) | |
| 1. **Clinical outcome**** | |  | |
| - Death | | 142 (1.9%) | |

* People without past medical history were assumed to have no comorbidity.

** Outcomes were censored on February 15th, 2021, regarding Center for Health Practice (CHP).

Supplementary Table 2. Demographics, clinical characteristics, and clinical outcomes of validation set

| **Patients Characteristics (*n* = 722)** | **Median (IQR) or Count (%)** | **Missing Count (%)** |
| --- | --- | --- |
| 1. **Demographics** |  |  |
| Age (years) | 43 (32-56) | 3 (0.4%) |
| Sex (Males) | 368 (51.0%) | 3 (0.4%) |
| 1. **Complete Blood Count** |  |  |
| **White Blood Cell count** (*10^9^/L) | 5.8 (4.6-7.2) | 43 (6.0%) |
| Neutrophil count (*10^9^/L) | 2.6 (2.3-3.8) | 710 (98.3%) |
| Lymphocyte count (*10^9^/L) | 1.1 (0.8-1.4) | 710 (98.3%) |
| Monocyte count (*10^9^/L) | 0.5 (0.4-0.6) | 710 (98.3%) |
| Haemoglobin (g/dL) | 13.6 (12.5-14.6) | 43 (6.0%) |
| Haematocrit (L/L) | 0.40 (0.37-0.43) | 43 (6.0%) |
| Platelet (*10^9^/L) | 255 (200-316) | 79 (10.9%) |
| 1. **Liver function** |  |  |
| Albumin (g/L) | - | 722 (100.0%) |
| Total bilirubin (µmol/L) | - | 722 (100.0%) |
| Alanine aminotransferase (µ/L) | 27.0 (18.0-46.0) | 119 (16.5%) |
| Alkaline phosphatase (µ/L) | 69 (57-82) | 119 (16.5%) |
| 1. **Kidney function** |  |  |
| Urea (mmol/L) | 3.9 (3.2-4.8) | 95 (13.2%) |
| Creatinine (µmol/L) | - | 722 (100.0%) |
| 1. **Inflammatory marker** |  |  |
| C-reactive protein | 0.8 (0.3-2.8) | 601 (83.2%) |
| 1. **Electrolyte** |  |  |
| Sodium (mmol/L) | 138 (136-139) | 669 (92.7%) |
| Potassium (mmol/L) | 3.7 (3.4-4.0) | 669 (92.7%) |
| Phosphate (mmol/L) | - | 722 (100.0%) |
| Calcium (mmol/L) | - | 722 (100.0%) |
| 1. **Others** |  |  |
| Lactate dehydrogenase (µ/L) | 210.5 (173.9-263.1) | 127 (17.6%) |
| Creatine kinase (µ/L) | 95 (62-161) | 361 (50.0%) |
| 1. **Clinical outcome***** |  |  |
| Death | 8 (1.1%) | 0 (0.0%) |

*** Outcomes were censored on March 31th, 2021, regarding Center for Health Practice (CHP).

Supplementary Table 3. Comorbidities and clinical outcomes of four clusters

| **Characteristics** | **Cluster 1**  **(*n* = 1959)** | **Cluster 2**  **(*n* = 2224)** | **Cluster 3**  **(*n* = 1850)** | **Cluster 4**  **(*n* = 1573)** | **p value** |
| --- | --- | --- | --- | --- | --- |
|  | **Median (IQR) or Count (% within cluster)** | | | |  |
| 1. **Demographics** |  |  |  |  |  |
| Age (years) | 36a****  (24-50) | 38b  (27-53) | 51c  (38-61) | 65d  (57-75) | <0.001 |
| Sex (Males) | 285a  (14.5%) | 1947b  (87.5%) | 442c  (23.9%) | 1023d  (65.0%) |  |
| 1. **Comorbidities** |  |  |  |  |  |
| Any | 1355 (69.2%)a | 1517 (68.2%)a | 1424 (77.0%)b | 1391 (88.4%)c | <0.001 |
| Infectious and Parasitic Diseases | 481 (24.6%)a | 464 (20.9%)b | 428 (23.1%)a, b | 408 (25.9%)c | <0.001 |
| Neoplasms | 77 (3.9%)a | 88 (4.0%)a | 179 (9.7%)b | 199 (12.7%)b | <0.001 |
| Endocrine, Nutritional and Metabolic Diseases, and Immunity Disorders | 222 (11.3%)a | 227 (10.2%)a | 281 (15.2%)b | 666 (42.3%)c | <0.001 |
| Diseases of the Blood and Blood-forming Organs | 101 (5.2%)a | 30 (1.3%)b | 109 (5.9%)a | 103 (6.5%)a | <0.001 |
| Mental Disorders | 121 (6.2%)a | 156 (7.0%)a | 194 (10.5%)b | 211 (13.4%)c | <0.001 |
| Diseases of the Nervous System and Sense Organs | 185 (9.4%)a | 211 (9.5%)a | 245 (13.2%)b | 402 (25.6%)c | <0.001 |
| Diseases of the Circulatory System | 211 (10.8%)a | 273 (12.3%)a | 328 (17.7%)b | 753 (47.9%)c | <0.001 |
| Diseases of the Respiratory System | 639 (32.6%)a | 722 (32.5%)a | 602 (32.5%)a | 650 (41.3%)c | <0.001 |
| Diseases of the Digestive System | 318 (16.2%)a | 381 (17.1%)a, b | 373 (20.2%)b | 528 (33.6%)c | <0.001 |
| Diseases of the Genitourinary System | 260 (13.3%)a | 194 (8.7%)b | 357 (19.3%)c | 425 (27.0%)d | <0.001 |
| Complications of Pregnancy, Childbirth, and the Puerperium | 297 (15.2%)a | 42 (1.9%)b | 296 (16.0%)a | 46 (2.9%)b | <0.001 |
| Diseases of the Skin and Subcutaneous Tissue | 123 (6.3%)a | 162 (7.3%)a | 148 (8.0%)a | 220 (14.0%)b | <0.001 |
| Diseases of the Musculoskeletal System and Connective Tissue | 214 (10.9%)a | 281 (12.6%)a | 309 (16.7%)b | 396 (25.2%)c | <0.001 |
| Congenital Anomalies | 51 (2.6%)a | 36 (1.6%)a, b | 26 (1.4%)a, b | 16 (1.0%)b | 0.002 |
| Certain Conditions originating in the Perinatal Period | 115 (5.9%)a | 59 (2.7%)b | 12 (0.6%)c | 1 (0.1%)d | <0.001 |
| Symptoms, Signs and Ill-defined Conditions | 614 (31.3%)a | 613 (27.6%)b | 707 (38.2%)c | 845 (53.7%)d | <0.001 |
| Injury and Poisoning | 417 (21.3%)a | 682 (30.7%)b | 497 (26.9%)c | 599 (38.1%)d | <0.001 |
| Infectious and Parasitic Diseases | 523 (26.7%)a | 368 (16.5%)b | 531 (28.7%)a | 482 (30.6%)a | <0.001 |
| Neoplasms | 373 (19.0%)a | 585 (26.3%)b | 428 (23.1%)b | 543 (34.5%)c | <0.001 |
| 1. **Clinical outcome** |  |  |  |  |  |
| Death | 4 (0.2%)a | 6 (0.3%)a | 9 (0.5%)a | 123 (7.8%)b |  |
| (% of Total Death) | (2.8%) | (4.2%) | (6.3%) | (86.6%) |  |

****The clusters with different letter are significantly different from each other at the 0.05 significance level.
